# Supplementary material for: Successful mating and hybridisation in two closely related flatworm species despite significant differences in reproductive morphology and behaviour
Source: Sci Rep. 2020 Jul 30;10:12830. doi: 10.1038/s41598-020-69767-5 (PMC7393371; doi:10.1038/s41598-020-69767-5)
Supplement: Supplementary file 1 — Supplementary Information. [file 41598_2020_69767_MOESM1_ESM.docx]

***Successful mating and hybridisation in two closely related flatworm species despite significant differences in reproductive morphology and behaviour***

Pragya Singh^*1^, Daniel N. Ballmer^1,2^, Max Laubscher^1^ and Lukas Schärer^1^

^1^University of Basel, Department of Environmental Sciences, Zoological Institute, Basel, Switzerland

^2^Friedrich Miescher Institute for Biomedical Research, Basel, Switzerland

^*^Corresponding Author:

Email: [pragya.singh42019@gmail.com](mailto:pragya.singh42019@gmail.com)

ORCiD: https://orcid.org/0000-0002-7411-3206

University of Basel, Department of Environmental Sciences, Zoological Institute, Vesalgasse 1, 4051 Basel, Switzerland

Supplementary Table S1. No. of offspring produced while paired and while in isolation from F1 hybrid x F1 hybrid pairings.

| pair no. | Number of hatchlings while paired for 7 days | Number of hatchlings from hybrid 1 while in isolation for 14 days | Number of hatchlings from hybrid 2 while in isolation for 14 days |
| --- | --- | --- | --- |
| 1 | 3 | 7 | 0 |
| 2 | 1 | 0 | 1 |
| 3 | 6 | 2 | 4 |
| 4 | 4 | 0 | 5 |
| 5 | 9 | 0 | 5 |
| 6 | 8 | 0 | 4 |
| 7 | 5 | 0 | 0 |
| 8 | 0 | 0 | 0 |
| 9 | 7 | 3 | 0 |
| 10 | 6 | 0 | 5 |
| 11 | 4 | 0 | 2 |
| 12 | 4 | 0 | 3 |
| 13 | 5 | 4 | 1 |
| 14 | 3 | 0 | 5 |
| 15 | 10 | 6 | 1 |
| 16 | 8 | 0 | 0 |
| 17 | 2 | 0 | 4 |
| 18 | 5 | 0 | 5 |
| 19 | 7 | 0 | 0 |
| 20 | 5 | 0 | 0 |
| 21 | 1 | 0 | 0 |
| 22 | 4 | 0 | 4 |
| 23 | 8 | 4 | 4 |
| 24 | 9 | 0 | 6 |
| 25 | 2 | 2 | 0 |

Supplementary Figure S2. Effect of dye on mating rate of a) *M. lignano* and b) *M. janickei*.


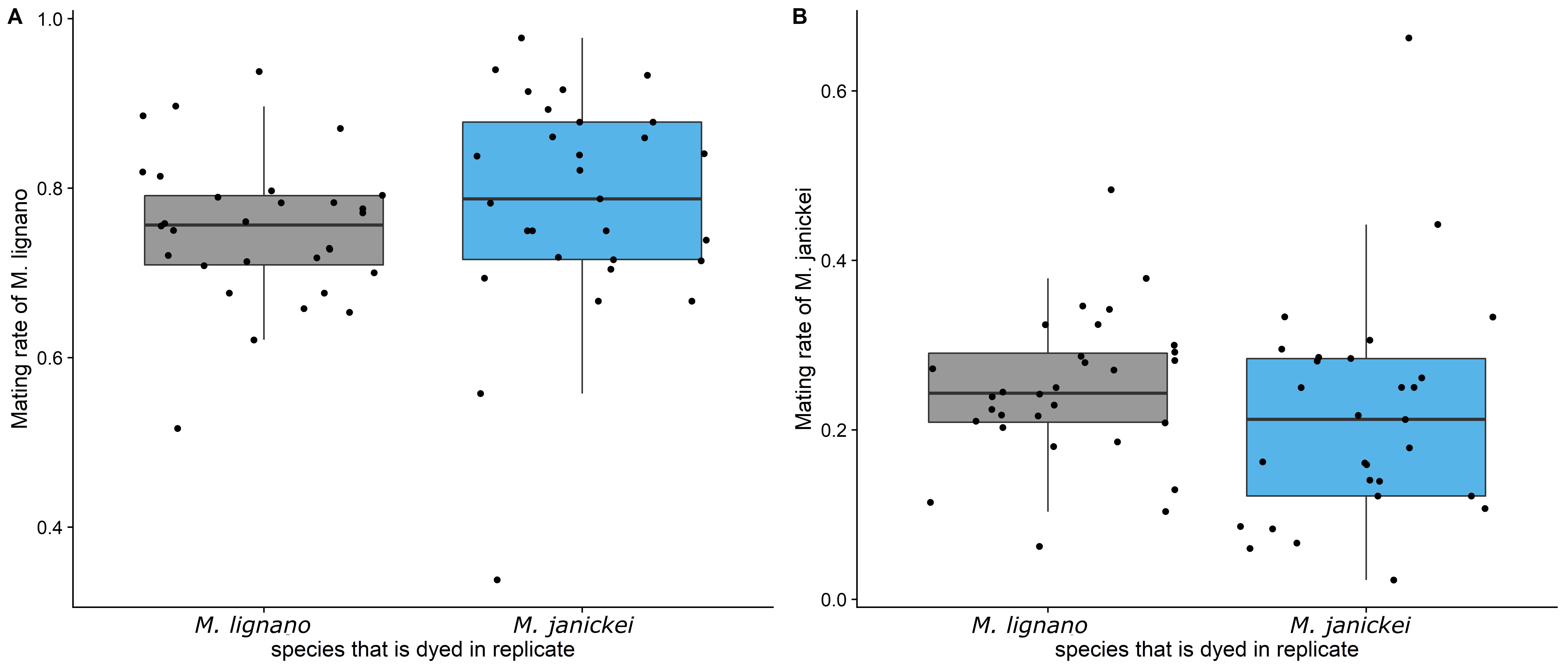


Welch Two Sample t-test:

Effect of dye on *M. lignano* (comparing replicates in which *M. lignano* was dyed with replicates in which *M. lignano* was not dyed)

t = -1.0923, df = 48.152, p-value = 0.2801

We only test the effect of dye on mating rate of *M. lignano*. We get same results for *M. janickei* as its mating rate = 1-mating rate of *M. lignano.*

Supplementary Table S3. Number of copulations of different types for each replicate, and the associated G-tests of goodness-of-fit with both uncorrected P-values and P-values after Bonferroni correction for multiple testing.

| Movie | Drop no. | Total no. of copulations in drop | No. of copulations of *M. lignano* conspecific type | No. of copulations of heterospecific type | No. of copulations of *M. janickei* conspecific type | G | df | P | P  (corrected for multiple testing) |
| --- | --- | --- | --- | --- | --- | --- | --- | --- | --- |
| A | 1 | 20 | 13 | 4 | 3 | 14.45 | 2 | 0.001 | 0.043 |
| A | 2 | 14 | 9 | 5 | 0 | 13.64 | 2 | 0.001 | 0.065 |
| A | 3 | 44 | 32 | 10 | 2 | 45.75 | 2 | <0.001 | <0.001 |
| A | 4 | 39 | 21 | 14 | 4 | 15.82 | 2 | <0.001 | 0.022 |
| C | 1 | 35 | 17 | 15 | 3 | 11.53 | 2 | 0.003 | 0.185 |
| C | 2 | 49 | 28 | 12 | 9 | 23.61 | 2 | <0.001 | <0.001 |
| C | 3 | 40 | 5 | 17 | 18 | 8.70 | 2 | 0.013 | 0.760 |
| C | 4 | 59 | 34 | 17 | 8 | 28.26 | 2 | <0.001 | <0.001 |
| D | 1 | 14 | 6 | 8 | 0 | 8.60 | 2 | 0.014 | 0.799 |
| D | 2 | 61 | 28 | 31 | 2 | 26.91 | 2 | <0.001 | <0.001 |
| D | 3 | 48 | 27 | 20 | 1 | 31.53 | 2 | <0.001 | <0.001 |
| E | 1 | 33 | 13 | 15 | 5 | 3.96 | 2 | 0.138 | 1.000 |
| E | 2 | 40 | 23 | 17 | 0 | 32.79 | 2 | <0.001 | <0.001 |
| E | 3 | 26 | 8 | 13 | 5 | 0.70 | 2 | 0.705 | 1.000 |
| E | 4 | 38 | 18 | 14 | 6 | 8.94 | 2 | 0.011 | 0.675 |
| F | 1 | 38 | 26 | 8 | 4 | 31.59 | 2 | <0.001 | <0.001 |
| F | 2 | 18 | 8 | 8 | 2 | 4.08 | 2 | 0.130 | 1.000 |
| F | 3 | 33 | 15 | 14 | 4 | 7.54 | 2 | 0.023 | 1.000 |
| F | 4 | 39 | 21 | 9 | 9 | 16.87 | 2 | <0.001 | 0.013 |
| G | 1 | 48 | 29 | 14 | 5 | 27.33 | 2 | <0.001 | <0.001 |
| G | 3 | 32 | 20 | 11 | 1 | 24.25 | 2 | <0.001 | <0.001 |
| G | 4 | 32 | 21 | 4 | 7 | 27.57 | 2 | <0.001 | <0.001 |
| H | 1 | 31 | 17 | 13 | 1 | 18.04 | 2 | <0.001 | 0.007 |
| H | 2 | 44 | 25 | 15 | 4 | 21.47 | 2 | <0.001 | 0.001 |
| H | 4 | 68 | 33 | 33 | 2 | 33.25 | 2 | <0.001 | <0.001 |
| I | 1 | 37 | 25 | 12 | 0 | 39.32 | 2 | <0.001 | <0.001 |
| I | 2 | 30 | 22 | 3 | 5 | 33.64 | 2 | <0.001 | <0.001 |
| I | 3 | 54 | 31 | 11 | 12 | 28.96 | 2 | <0.001 | <0.001 |
| I | 4 | 25 | 22 | 3 | 0 | 46.81 | 2 | <0.001 | <0.001 |
| J | 1 | 44 | 23 | 17 | 4 | 17.07 | 2 | <0.001 | 0.012 |
| J | 3 | 54 | 26 | 21 | 7 | 14.33 | 2 | 0.001 | 0.046 |
| J | 4 | 15 | 14 | 0 | 1 | 34.24 | 2 | <0.001 | <0.001 |
| K | 1 | 72 | 46 | 26 | 0 | 69.40 | 2 | <0.001 | <0.001 |
| K | 3 | 43 | 31 | 12 | 0 | 51.67 | 2 | <0.001 | <0.001 |
| K | 4 | 29 | 23 | 6 | 0 | 42.52 | 2 | <0.001 | <0.001 |
| L | 1 | 46 | 29 | 12 | 5 | 29.70 | 2 | <0.001 | <0.001 |
| L | 2 | 24 | 20 | 4 | 0 | 39.37 | 2 | <0.001 | <0.001 |
| L | 3 | 41 | 31 | 10 | 0 | 54.26 | 2 | <0.001 | <0.001 |
| L | 4 | 30 | 9 | 13 | 8 | 0.59 | 2 | 0.743 | 1.000 |
| M | 1 | 22 | 11 | 9 | 2 | 7.59 | 2 | 0.022 | 1.000 |
| M | 2 | 23 | 16 | 4 | 3 | 20.40 | 2 | <0.001 | 0.002 |
| M | 3 | 47 | 27 | 17 | 3 | 25.73 | 2 | <0.001 | <0.001 |
| M | 4 | 23 | 17 | 2 | 4 | 26.96 | 2 | <0.001 | <0.001 |
| N | 1 | 35 | 28 | 6 | 1 | 47.95 | 2 | <0.001 | <0.001 |
| N | 2 | 20 | 13 | 4 | 3 | 14.45 | 2 | 0.001 | 0.043 |
| N | 3 | 41 | 32 | 8 | 1 | 53.15 | 2 | <0.001 | <0.001 |
| N | 4 | 29 | 17 | 11 | 1 | 18.94 | 2 | <0.001 | 0.005 |
| O | 1 | 22 | 21 | 1 | 0 | 51.47 | 2 | <0.001 | <0.001 |
| O | 2 | 35 | 23 | 11 | 1 | 29.90 | 2 | <0.001 | <0.001 |
| O | 3 | 8 | 7 | 1 | 0 | 14.77 | 2 | 0.001 | 0.037 |
| O | 4 | 28 | 22 | 6 | 0 | 40.22 | 2 | <0.001 | <0.001 |
| P | 1 | 29 | 25 | 3 | 1 | 48.48 | 2 | <0.001 | <0.001 |
| P | 2 | 24 | 12 | 10 | 2 | 8.59 | 2 | 0.014 | 0.803 |
| P | 3 | 24 | 12 | 11 | 1 | 11.14 | 2 | 0.004 | 0.225 |
| P | 4 | 44 | 25 | 16 | 3 | 23.06 | 2 | <0.001 | 0.001 |
| Q | 1 | 54 | 41 | 12 | 1 | 66.42 | 2 | <0.001 | <0.001 |
| Q | 2 | 32 | 24 | 7 | 1 | 37.00 | 2 | <0.001 | <0.001 |
| Q | 3 | 28 | 21 | 5 | 2 | 30.83 | 2 | <0.001 | <0.001 |
| Q | 4 | 36 | 23 | 11 | 2 | 26.31 | 2 | <0.001 | <0.001 |

Supplementary Table S4. Observed and expected number of copulations of different types and mating rate of each species for each replicate, and the associated Chi-square goodness-of-fit test with both uncorrected P-values and P-values after Bonferroni correction for multiple testing.

| Movie name | Drop no. | Total no. of copulations in drop of *M. lignano* | Total no. of copulations in drop of *M. janickei* | observed mating rate of *M. lignano* (p) | observed mating rate of *M. janickei* (q) | Expected no. of copulations of *M. lignano* conspecific type | Expected no. of copulations of heterospecific type | Expected no. of copulations of *M. janickei* conspecific type | chi-square | df | P | P  (corrected for multiple testing) |
| --- | --- | --- | --- | --- | --- | --- | --- | --- | --- | --- | --- | --- |
| A | 1 | 30 | 10 | 0.75 | 0.25 | 11.25 | 7.5 | 1.25 | 4.36 | 2 | 0.11 | 1 |
| A | 2 | 23 | 5 | 0.82 | 0.18 | 9.45 | 4.11 | 0.45 | 0.66 | 2 | 0.72 | 1 |
| A | 3 | 74 | 14 | 0.84 | 0.16 | 31.11 | 11.77 | 1.11 | 1 | 2 | 0.61 | 1 |
| A | 4 | 56 | 22 | 0.72 | 0.28 | 20.1 | 15.79 | 3.1 | 0.5 | 2 | 0.78 | 1 |
| C | 1 | 49 | 21 | 0.7 | 0.3 | 17.15 | 14.7 | 3.15 | 0.01 | 2 | 0.99 | 1 |
| C | 2 | 68 | 30 | 0.69 | 0.31 | 23.59 | 20.82 | 4.59 | 8.79 | 2 | 0.01 | 0.73 |
| C | 3 | 27 | 53 | 0.34 | 0.66 | 4.56 | 17.89 | 17.56 | 0.1 | 2 | 0.95 | 1 |
| C | 4 | 85 | 33 | 0.72 | 0.28 | 30.61 | 23.77 | 4.61 | 4.79 | 2 | 0.09 | 1 |
| D | 1 | 20 | 8 | 0.71 | 0.29 | 7.14 | 5.71 | 1.14 | 2.24 | 2 | 0.33 | 1 |
| D | 2 | 87 | 35 | 0.71 | 0.29 | 31.02 | 24.96 | 5.02 | 3.57 | 2 | 0.17 | 1 |
| D | 3 | 74 | 22 | 0.77 | 0.23 | 28.52 | 16.96 | 2.52 | 1.54 | 2 | 0.46 | 1 |
| E | 1 | 41 | 25 | 0.62 | 0.38 | 12.73 | 15.53 | 4.73 | 0.04 | 2 | 0.98 | 1 |
| E | 2 | 63 | 17 | 0.79 | 0.21 | 24.81 | 13.39 | 1.81 | 2.91 | 2 | 0.23 | 1 |
| E | 3 | 29 | 23 | 0.56 | 0.44 | 8.09 | 12.83 | 5.09 | 0 | 2 | 1 | 1 |
| E | 4 | 50 | 26 | 0.66 | 0.34 | 16.45 | 17.11 | 4.45 | 1.25 | 2 | 0.53 | 1 |
| F | 1 | 60 | 16 | 0.79 | 0.21 | 23.68 | 12.63 | 1.68 | 5.11 | 2 | 0.08 | 1 |
| F | 2 | 24 | 12 | 0.67 | 0.33 | 8 | 8 | 2 | 0 | 2 | 1 | 1 |
| F | 3 | 44 | 22 | 0.67 | 0.33 | 14.67 | 14.67 | 3.67 | 0.07 | 2 | 0.97 | 1 |
| F | 4 | 51 | 27 | 0.65 | 0.35 | 16.67 | 17.65 | 4.67 | 9.37 | 2 | 0.01 | 0.54 |
| G | 1 | 72 | 24 | 0.75 | 0.25 | 27 | 18 | 3 | 2.37 | 2 | 0.31 | 1 |
| G | 3 | 51 | 13 | 0.8 | 0.2 | 20.32 | 10.36 | 1.32 | 0.12 | 2 | 0.94 | 1 |
| G | 4 | 46 | 18 | 0.72 | 0.28 | 16.53 | 12.94 | 2.53 | 15.27 | 2 | 0.0004 | 0.03 |
| H | 1 | 47 | 15 | 0.76 | 0.24 | 17.81 | 11.37 | 1.81 | 0.64 | 2 | 0.73 | 1 |
| H | 2 | 65 | 23 | 0.74 | 0.26 | 24.01 | 16.99 | 3.01 | 0.6 | 2 | 0.74 | 1 |
| H | 4 | 99 | 37 | 0.73 | 0.27 | 36.03 | 26.93 | 5.03 | 3.45 | 2 | 0.18 | 1 |
| I | 1 | 62 | 12 | 0.84 | 0.16 | 25.97 | 10.05 | 0.97 | 1.39 | 2 | 0.5 | 1 |
| I | 2 | 47 | 13 | 0.78 | 0.22 | 18.41 | 10.18 | 1.41 | 14.93 | 2 | 0.0006 | 0.03 |
| I | 3 | 73 | 35 | 0.68 | 0.32 | 24.67 | 23.66 | 5.67 | 15.46 | 2 | 0.0004 | 0.03 |
| I | 4 | 47 | 3 | 0.94 | 0.06 | 22.09 | 2.82 | 0.09 | 0.1 | 2 | 0.95 | 1 |
| J | 1 | 63 | 25 | 0.72 | 0.28 | 22.55 | 17.9 | 3.55 | 0.11 | 2 | 0.95 | 1 |
| J | 3 | 73 | 35 | 0.68 | 0.32 | 24.67 | 23.66 | 5.67 | 0.68 | 2 | 0.71 | 1 |
| J | 4 | 28 | 2 | 0.93 | 0.07 | 13.07 | 1.87 | 0.07 | 15 | 2 | 0.0005 | 0.03 |
| K | 1 | 118 | 26 | 0.82 | 0.18 | 48.35 | 21.31 | 2.35 | 3.5 | 2 | 0.17 | 1 |
| K | 3 | 74 | 12 | 0.86 | 0.14 | 31.84 | 10.33 | 0.84 | 1.13 | 2 | 0.57 | 1 |
| K | 4 | 52 | 6 | 0.9 | 0.1 | 23.31 | 5.38 | 0.31 | 0.39 | 2 | 0.82 | 1 |
| L | 1 | 70 | 22 | 0.76 | 0.24 | 26.63 | 16.74 | 2.63 | 3.69 | 2 | 0.16 | 1 |
| L | 2 | 44 | 4 | 0.92 | 0.08 | 20.17 | 3.67 | 0.17 | 0.2 | 2 | 0.91 | 1 |
| L | 3 | 72 | 10 | 0.88 | 0.12 | 31.61 | 8.78 | 0.61 | 0.79 | 2 | 0.67 | 1 |
| L | 4 | 31 | 29 | 0.52 | 0.48 | 8.01 | 14.98 | 7.01 | 0.53 | 2 | 0.77 | 1 |
| M | 1 | 31 | 13 | 0.7 | 0.3 | 10.92 | 9.16 | 1.92 | 0.01 | 2 | 1 | 1 |
| M | 2 | 36 | 10 | 0.78 | 0.22 | 14.09 | 7.83 | 1.09 | 5.5 | 2 | 0.06 | 1 |
| M | 3 | 71 | 23 | 0.76 | 0.24 | 26.81 | 17.37 | 2.81 | 0.02 | 2 | 0.99 | 1 |
| M | 4 | 36 | 10 | 0.78 | 0.22 | 14.09 | 7.83 | 1.09 | 12.75 | 2 | 0.002 | 0.1 |
| N | 1 | 62 | 8 | 0.89 | 0.11 | 27.46 | 7.09 | 0.46 | 0.82 | 2 | 0.66 | 1 |
| N | 2 | 30 | 10 | 0.75 | 0.25 | 11.25 | 7.5 | 1.25 | 4.36 | 2 | 0.11 | 1 |
| N | 3 | 72 | 10 | 0.88 | 0.12 | 31.61 | 8.78 | 0.61 | 0.32 | 2 | 0.85 | 1 |
| N | 4 | 45 | 13 | 0.78 | 0.22 | 17.46 | 10.09 | 1.46 | 0.24 | 2 | 0.89 | 1 |
| O | 1 | 43 | 1 | 0.98 | 0.02 | 21.01 | 0.98 | 0.01 | 0.01 | 2 | 0.99 | 1 |
| O | 2 | 57 | 13 | 0.81 | 0.19 | 23.21 | 10.59 | 1.21 | 0.05 | 2 | 0.97 | 1 |
| O | 3 | 15 | 1 | 0.94 | 0.06 | 7.03 | 0.94 | 0.03 | 0.04 | 2 | 0.98 | 1 |
| O | 4 | 50 | 6 | 0.89 | 0.11 | 22.32 | 5.36 | 0.32 | 0.4 | 2 | 0.82 | 1 |
| P | 1 | 53 | 5 | 0.91 | 0.09 | 24.22 | 4.57 | 0.22 | 3.42 | 2 | 0.18 | 1 |
| P | 2 | 34 | 14 | 0.71 | 0.29 | 12.04 | 9.92 | 2.04 | 0 | 2 | 1 | 1 |
| P | 3 | 35 | 13 | 0.73 | 0.27 | 12.76 | 9.48 | 1.76 | 0.62 | 2 | 0.73 | 1 |
| P | 4 | 66 | 22 | 0.75 | 0.25 | 24.75 | 16.5 | 2.75 | 0.04 | 2 | 0.98 | 1 |
| Q | 1 | 94 | 14 | 0.87 | 0.13 | 40.91 | 12.19 | 0.91 | 0.01 | 2 | 0.99 | 1 |
| Q | 2 | 55 | 9 | 0.86 | 0.14 | 23.63 | 7.73 | 0.63 | 0.29 | 2 | 0.87 | 1 |
| Q | 3 | 47 | 9 | 0.84 | 0.16 | 19.72 | 7.55 | 0.72 | 3.2 | 2 | 0.2 | 1 |
| Q | 4 | 57 | 15 | 0.79 | 0.21 | 22.56 | 11.88 | 1.56 | 0.2 | 2 | 0.91 | 1 |
